# Review article: Use of prehospital early warning scores to predict short‐term mortality: A systematic review

**DOI:** 10.1111/1742-6723.70047

**Published:** 2025-05-02

**Authors:** David Naylor, Bridget Dicker, Graham Howie, Verity Todd

**Affiliations:** ^1^ Paramedicine Research Unit, Paramedicine Department, Faculty of Health and Environmental Sciences Auckland University of Technology Auckland New Zealand; ^2^ Clinical Audit and Research Team Hato Hone St John Auckland New Zealand

**Keywords:** early warning score, emergency service, mortality, out of hospital, paramedic, prehospital

## Abstract

Early Warning Scores (EWS) have been developed to identify patients at risk of deterioration. Although the application of EWS has become increasingly established in the prehospital setting, their use remains contentious. The aim of this systematic review is to summarise the most recent evidence on the predictive accuracy of the EWS for short‐term mortality in adults in the prehospital setting. A systematic search was conducted using the Medline, CINAHL, and Scopus databases. Studies that evaluated the diagnostic accuracy of the prehospital Modified Early Warning Score, National Early Warning Score or National Early Warning Score 2 in predicting mortality were included. Secondary outcomes were intensive care unit (ICU) admission and hospital admission. The review included 16 studies published between 2012 and 2023, with the number of patients totalling 311 932. The literature indicated that prehospital EWS demonstrated a moderate to good diagnostic performance in predicting short‐term mortality with an area under receiver operating characteristic curve ranging from 0.68 (95% confidence interval [CI]: 0.64–0.73) to 0.90 (95% CI: 0.82–0.97). Overall, diagnostic performance was higher for predicting mortality in short time frames (up to 48 h). The need to use relatively high cut‐off points to identify at‐risk patients may limit its use for the unselected patient populations found in the prehospital setting. The potential for under‐triage and over‐triage limits their use further. EWS should not replace structured clinical evaluation and judgement but may be useful as complementary and objective tools to aid the identification of patients at risk.

## Graphical abstract

The prehospital use of Early Warning Scores (EWS) has a moderate to good diagnostic accuracy in predicting critical illness. Prehospital EWS should not replace structured clinical assessment and judgement but can be useful as complementary and objective tools to aid the identification of patients at risk of deterioration.

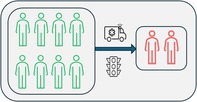

**Key findings**

- Use of Early Warning Scores (MEWS, NEWS, NEWS2) in the prehospital setting has a moderate to good diagnostic accuracy in predicting short‐term mortality in adults, but not ICU admission.
- The use of high cut‐off points and the potential for over‐triage and under‐triage may suggest that prehospital Early Warning Score use is limited.
- Early Warning Scores should not replace structured clinical assessment and judgement but can be useful as complementary tools to aid in the identification of patients at risk.

## Introduction

A priority of the healthcare system is the early identification of high‐risk patients. Early identification of critical illness can reduce the time to definitive treatment and improve patient outcomes.^1^ Critical illness is a state of severity and although a consensus definition has not been established, there are common themes across published studies which include vital organ dysfunction and imminent death.^2^ Ambulance paramedics are often the first point of contact for these patients and are required to make time‐critical clinical decisions based on limited information and diagnostic tools.^3^ Paramedic decision‐making is highly contextual and requires flexibility to account for the unpredictable environment in which they work.^4^ However, this dynamism and unpredictability can lead to a high level of subjectivity and the practice of inadvertently overlooking clinical deterioration in the prehospital setting has been well‐documented.^5^, ^6^ Early Warning Scores (EWS) have been developed to identify patients at risk of deterioration, ensure objective clinical decision‐making and guide appropriate clinical interventions.^7^ Therefore, the use of EWS in the prehospital setting may facilitate the earlier identification of at‐risk patients.

Initially developed for use in intensive care units (ICUs), EWS systems have been utilised in various healthcare settings including hospital wards, the ED, and, more recently, in the prehospital setting.^8^ Although modifications to the original EWS have been made to suit these different settings and, in some cases, particular illnesses, the fundamental principles have remained the same.^9^ These clinical prediction models assign numerical weighting to defined clinical measurements such as level of consciousness, blood pressure, heart rate and oxygen saturation. The composite score, or a more significant change in a single parameter, is linked to predefined triggers that initiate review and treatment.^7^ The Modified Early Warning Score (MEWS) was introduced in the United Kingdom (UK) to address some of the limitations of the original EWS, adding slight adjustments to scoring thresholds and parameters.^10^

The development of different regional EWS systems resulted in a lack of consistency in the recognition of and response to clinical deterioration.^11^ The National Early Warning Score (NEWS) was developed in the UK by the Royal College of Physicians in 2012 with the aim of standardising these various approaches to EWS.^12^ The NEWS was updated in 2017 to incorporate new‐onset confusion, recognised as an important sign of clinical deterioration, and adjustments for patients with long‐term hypercapnic respiratory failure (NEWS2).^7^ The NEWS, NEWS2^13^ and the previously developed MEWS^10^ remain the most commonly used EWS in the ED and the prehospital setting.^8^

EWS have become an integral part of clinical practice in hospitals and healthcare settings globally.^8^ The use of EWS in hospital has been associated with improved patient outcomes, reduced morbidity, and reduced mortality rates.^14^ However, EWS are not widely used in the prehospital setting and their use remains contentious, with recent research suggesting a lower predictive accuracy in prehospital compared with in‐hospital use.^8^ The aim of this systematic review is to summarise the most recent evidence on the effectiveness and predictive accuracy of the prehospital use of MEWS, NEWS, and NEWS2 for mortality, ICU admission, and hospital admission.

## Methods

### Protocol and registration

The systematic review followed the preferred reporting items for systematic review and meta‐analysis (PRISMA) guidelines.^15^ The review was registered with the international register of systematic reviews (PROSPERO) on 26 August 2023 (CRD42023454212).

### Information sources and search strategy

A systematic literature search of the Ovid MEDLINE, ESBSCO CINAHL and Scopus databases was performed by the lead author on 28 August 2023. Key search terms were ‘Early Warning Score’ AND ‘prehospital’ OR ‘pre‐hospital’ OR ‘out of hospital’ OR ‘paramedic’ OR ‘ambulance’ OR ‘emergency services’ OR ‘emergency medical’. The reference lists of the included studies were also screened to identify additional studies.

### Eligibility criteria

Studies that evaluated the diagnostic accuracy of the MEWS, NEWS and NEWS2 in the prehospital setting were included, along with those that compared the performance of any of these EWS systems with current practice (non‐EWS). Only randomised controlled trials, case–control, cross‐sectional or cohort studies were included. Studies that limited the patient population to a particular age group or patient group (i.e., specific illness or injury) were excluded. Other exclusion criteria were paediatric patients (<15 years of age), pregnant patients, air transport involvement and inter‐facility transports. Paediatric patients and pregnant patients have different physiological responses to critical illness, and specific EWS have been developed for these populations.^16^, ^17^ Helicopter Emergency Services (HEMS) commonly attend to patients previously assessed and treated by other agencies, who are often more severely ill than the standard road patient cohort.^7^ This, alongside different patient recording systems, could distort findings of EWS application in the standard road‐based patient population.

Studies had to report data on mortality, with ICU admission and hospital admission considered as secondary outcomes.

### Study selection

Studies identified in the initial literature search were independently screened for eligibility, based on information contained in the title and abstract, by two reviewers (DN and VT) using the Rayyan software.^18^ All included and undecided studies were again reviewed by two reviewers (DN and VT), and any disagreement regarding eligibility was resolved by discussion.

### Data extraction

The lead author extracted data and collated the following information^1^: study characteristics: study location, study design, EWS type, study period, sample size, inclusion/exclusion criteria, EWS timing, patient age and gender (Table 1)^2^; study outcome: study outcomes, results (statistical analysis), conclusion (Table 2). A second author (VT) checked all input data.

**TABLE 1:** Study and patient characteristics

Study and country	Study design	EWS type	Study period	Sample size	Inclusion/Exclusion	EWS timing (set of vitals used in EWS calculation)	Patient demographic
Fullerton *et al*. (2012) UK	Retrospective observational	MEWS	April 2010 to June 2010	3057	Inclusion: >15 years old. All data recorded. Exclusion: Patients in cardiac arrest	1st set prehospital	Age (mean) 54.9 years Male 49.5%
Hoikka *et al*. (2018) Finland	Retrospective observational	NEWS	January 2014 to June 2014	12 426	Inclusion: >15 years old. Exclusion: Inter‐facility transport and homecare visits were excluded	1st set prehospital	Age (mean) 65.4 years Male 50.6%†
Lane *et al*. (2019) Canada	Retrospective observational	CIP, MEWS, NEWS	April 2015 to March 2016	121 837	Inclusion: >17 years old. Exclusion: Left ED without seeing a physician and inter‐hospital transfers	1st set prehospital	No mean or median given for the overall population.
Lindskou *et al*. (2023) Denmark	Retrospective observational	NEWS2, mNEWS, qSOFA, RETTS, DEPT	July 2016 to December 2020	107 569	Inclusion: >17 years using ambulance service. Exclusion: Death before record‐creation or at hospital arrival, no vital signs recorded	1st set prehospital, final set of prehospital, and worst (most severe)	Age (median) 65 years Male
Magnusson *et al*. (2020) Sweden	Prospective observational	RETT, NEWS, NEWS2	January 2016 to December 2016	4465	Inclusion: >15 years old. Assessed by EMS nurse. Exclusion: Patients who were not transported, interhospital transfers, assistance from other ambulances	On scene	Age (median) 69 years. Male 48%
Martin‐Rodriguez *et al*. (2019a) Spain	Prospective observational	EWS, NEWS2, MEWS, ViEWS, HEWS, SEWS	April 2018 to July 2018†	349	Inclusion: >18 years old. Transported by ALSU to reference hospital. Exclusion: Patient in cardiac arrest, pregnancy, psychiatric pathology, palliative care units, time of arrival of ALSU >45 min, discharge on scene	Timing unclear	Age (mean) 66.4 years. Male 58.5%
Martin‐Rodriguez *et al*. (2019b) Spain	Prospective observational	NEWS2	April 2018 to November 2018†	1054	Inclusion: >18 years old. Transported by ALSU to reference hospital. Exclusion: Patient in cardiac arrest, pregnancy, psychiatric pathology, palliative care units, time of arrival of ALSU >45 min, discharge on scene	Timing unclear	Age (median) 68 years Male 60.3%
Martin‐Rodriguez *et al*. (2019c) Spain	Prospective observational	NEWS2	April 2018 to February 2019†	1288	Inclusion: >18 years old. Transported by ALSU to reference hospital. Exclusion: Patient in cardiac arrest, pregnancy, psychiatric pathology, palliative care units, time of arrival of ALSU >45 min, discharge on scene	Timing unclear	Age (median) 68 years Male 59.5%
Martin‐Rodriguez *et al*. (2020) Spain	Prospective observational	NEWS2	March 2018 to May 2019†	2335	Inclusion: >18 years old. Transported by ALSU to reference hospital. Exclusion: Patient in cardiac arrest, pregnancy, psychiatric pathology, palliative care units, time of arrival of ALSU >45 min, discharge on scene	1st set prehospital	Age (median) 69 years. Male 58.9%
Martin‐Rodriguez *et al*. (2021) Spain	Prospective observational	NEWS2, MEWS, ViEWS, WPSS, TEWS, MREMS, PI	October 2018 to December 2019†	3273	Inclusion: >18 years old. Transported by ALSU to reference hospital. Exclusion: Patient in cardiac arrest, pregnancy, psychiatric pathology, terminally ill, high‐risk scenes, injuries incompatible with life, time of arrival of ALSU >45 min, discharge on scene	1st set prehospital	Age (median) 69 years Male 58.9%
Martin‐Rodriguez *et al*. (2023) Spain	Prospective observational	NEWS2	October 2018 to May 2021†	4943	Inclusion: >18 years old. Assessed by ALSU to reference hospital. Exclusion: Patient in cardiac arrest, pregnancy, psychiatric pathology, end‐of‐life care, high‐risk scenes, time of arrival of ALSU >45 min, discharge on scene	1st set prehospital, on evacuation, arrival at hospital	Age (median) 69 years Male 58.4%
Pirneskoski *et al*. (2019) Finland	Retrospective observational	NEWS	17 August 2008 to 18 December 2015	35 800	Inclusion: >18 years old. Exclusion: Insufficient data to calculate NEWS	1st set prehospital	Age (mean) 65.8 years Male 47.5%
Ruan *et al*. (2016) China	Prospective observational	MEWS	January 2013 to December 2014	10 517	Inclusion: >14 years old. Exclusion: Poor compliance (not specified), did not cooperate with diagnosis or treatment	On scene	Age (mean) 52.9 years Male 62.6%
Saberian *et al*. (2022) Iran	Prospective observational	NEWS	January 2021 to April 2021	1048	Inclusion: >15 years old, transported to ED *via* ambulance. Exclusion: Left ED against advice, transfers to other medical centres, missing triage data/lost follow‐up, death on scene or ED arrival	Timing unclear	Age (mean) 45.1 years Male 60%
Shaw *et al*. (2017) UK	Retrospective observational	NEWS	April 2012 to January 2013	287	Inclusion: > 15 years old, treated by ambulance clinicians, transported to hospital. Exclusion: Pregnancy, insufficient information to calculate NEWS	1st set prehospital, final set before ED arrival	Age (mean) 63 years Male 52%
Silcock *et al*. (2015) UK	Retrospective observational	NEWS	October 2012 to November 2012	1684	Inclusion: >15 years old. Exclusion: Pregnancy, transferred from other hospitals	Timing unclear	Age not available. Gender not available

† Hoikka *et al*. (2018): There is a potential error in the reporting of mean age and % male. Values reported in the demographics table were different from the in‐text values (Mean age 65.4 *vs* 63.1, % Male 50.6% *vs* 49.5%). Martin‐Rodriguez *et al*. 2019a, 2019b, 2019c, 2020, 2021, 2023 – these studies had an overlapping period. The findings are not independent of each other, as the same patients are included across all/most studies.

ALSU, advanced life support unit; CIP, critical illness prediction; DEPT, Danish emergency process triage; MEWS, Modified Early Warning Score; mNEWS, Modified National Early Warning Score; MREMS, Modified Rapid Emergency Medicine Score; NEWS, National Early Warning Score; NEWS2, National Early Warning Score 2; PI, Prehospital Index; TEWS, Triage Early Warning Score; ViEWS, Vitalpac Early Warning Score.

**TABLE 2:** Study outcomes

Study	EWS type	Outcomes and timing	Results	Optimal EWS threshold for predicting mortality	Study conclusions
Fullerton *et al*. (2012)	MEWS	Adverse event†: 24 h	Ambulance Clinical judgement‡	Not reported	Clinical judgement alone has low sensitivity (but high specificity) for critical illness (Prehospital). The addition of MEWS improves detection at the expense of specificity.
			Sensitivity 62% (95% CI, 51–73%), specificity 94% (95% CI 93–95%).		
			AUROC MEWS		
			0.80 (95% CI 0.74–0.86)		
Hoikka *et al*. (2018)	NEWS	Mortality: 1‐day, 30‐day	High‐risk NEWS group sensitivity and specificity	1‐day mortality: Increase in 1‐day mortality occurred with NEWS value >12	The high‐risk NEWS category was associated with 1‐day mortality well above the low‐ and medium‐risk NEWS categories. This effect was not as noticeable for 30‐day mortality. NEWS may be a useful tool in recognising pts. at early risk of death
			1‐day: 0.80 (95% CI: 0.74–0.86), 0.95 (CI 0.95–0.96)		
			30‐day: 0.42 (95% CI: 0.38–0.47), 0.96 (0.96–0.96)		
			Medium risk NEWS group – sensitivity and specificity:		
			1‐day: 0.89 (95% CI: 0.84–0.93), 0.81 (95% CI: 0.80–0.81)		
			30‐day: 0.63 (95% CI: 0.59–0.67), 0.82 (95% CI: 0.81–0.82)		
Lane *et al*. (2019)	CIP, MEWS, NEWS	Mortality: at hospital discharge, 48 h	AUROC:	Not reported	Prognostic scores using physiologic measures assessed by paramedics have good predictive ability for mortality.
			Hospital mortality: MEWS 0.71, NEWS 0.78		
			2‐day mortality: MEWS 0.80, NEWS 0.85		
			(Confidence interval not stated)		
Lindskou *et al*. (2023)	MEWS2, mNEWS, qSOFA, RETTS, DEPT	Mortality: 1‐day, 30‐day ICU admission	AUROC (NEWS2):	Not reported	EWS performed moderately in predicting short‐term mortality. At typical operating points there are high numbers of false negatives and false positives, suggesting a risk of under‐triage and over‐triage.
			1‐day mortality: 0.72 (95% CI: 0.71–0.73)		
			30‐day mortality: 0.68 (95% CI: 0.68–0.69)		
			ICU admission: 0.68 (95%: 0.67–0.69)		
Magnusson *et al*. (2020)	RETTS‐A, NEWS, NEWS2	Mortality: 48‐h, 30‐day Time‐sensitive condition (TSC)§	AUROC:	Not reported	RETTS‐A showed better sensitivity in detecting time‐sensitive conditions but with a lower specificity than NEWS and NEWS2. Field assessment was appropriate in the majority of cases (in terms of diagnosis) but a role was recognised for EWS in complex decision‐making.
			48‐h mortality: NEWS 0.75 (95% CI: 0.66–0.83), NEWS2 0.77 (95% CI: 0.68–0.86).		
			30‐day mortality: NEWS 0.70 (95% CI: 0.66–0.75). NEWS2 0.68 (95% CI: 0.64–0.73).		
			TSC AUROC:		
			NEWS: 0.58 (0.55–0.61), NEWS2: 0.59 (0.56–0.62)		
Martin‐Rodriguez *et al*. (2019a)	EWS, NEWS2, MEWS, ViEWS, HEWS, SEWS	Mortality: 48‐h	AUROC: 48‐h mortality: MEWS 0.85 (95% CI: 0.76–02.93), NEWS2 0.90 (95% CI: 0.82–0.95)	48‐h mortality: NEWS2 – 10 MEWS – 5	No significant statistical difference between the EWS studied, but NEWS2 was validated at the prehospital level and would complement the structured and objective evaluation of the critical patient
Martin‐Rodriguez *et al*. (2019b)	NEWS2	Mortality: 48 h, 7‐day, 30‐day	AUROC (NEWS2):	48‐h mortality: NEWS2 – between 7 and 9 depending on triage priority levels	Used within a structured assessment and triage system, NEWS2 helps to predict early mortality and detect high‐risk patients
			48‐h mortality: 0.88 (95% CI: 0.82–0.94)		
			7‐day mortality: 0.86 (CI 95%: 0.81–0.91)		
			30‐day mortality: 0.82 (95% CI: 0.77–0.87)		
Martin‐Rodriguez *et al*. (2019c)	NEWS2	Mortality: 48‐h	AUROC:	48‐h mortality:	NEWS2 scores are easy to obtain and help in the initial assessment of high‐risk patients.
			48‐h mortality: 0.89 (95% CI: 0.84–0.94)	NEWS2 – 9	
Martin‐Rodriguez *et al*. (2020)	NEWS2	Mortality: 1‐day, 2‐day, 7‐day, 30‐day	AUROC (NEWS2):	48‐h mortality:	NEWS2 performed well at the prehospital level in predicting early mortality.
			1‐day mortality: 0.86 (95% CI: 0.78–0.93)	NEWS2 – 9	
			2‐day mortality: 0.89 (95% CI: 0.84–0.92)		
			7‐day mortality: 0.84 (95% CI: 0.79–0.87)		
			30‐day mortality: 0.81 (95% CI: 0.77–0.84)		
Martin‐Rodriguez *et al*. (2021)	NEWS2, MEWS, ViEWS, WPSS, TEWS, MREMS, PI	Mortality: 1‐day, 2‐day, 7‐day	AUROC:	1‐day mortality: NEWS – 7 MEWS – 5	All scores have a good predictive capacity for early mortality. No statistically significant differences in the performance.
			1‐day mortality: MEWS 0.85 (0.8–0.89), NEWS2 0.86 (95% CI: 0.81–0.90) 2‐day mortality: MEWS 0.85 (95% CI: 0.80–0.88), NEWS2 0.86 (95% CI: 0.82–0.89)		
			3‐day mortality: MEWS 0.83 (95% CI: 0.79–0.86), NEWS2 0.84 (95% CI: 0.80–0.88)		
			7‐day mortality: MEWS 0.79 (95% CI: 0.75–0.82) NEWS2 0.82 (95% CI: 0.78–0.85)		
Martin‐Rodriguez *et al*. (2023)	NEWS2	Mortality: 2‐day ICU admission	AUROC (NEWS2, on scene – baseline):	Not reported	NEWS2 has good performance for both outcomes. Very consistent response over the time
			2‐day mortality: 0.87 (95% CI: 0.83–0.91)		
			ICU admission: 0.76 (95% CI: 0.72–0.80)		
Pirneskoski *et al*. (2019)	NEWS	Mortality: 24‐h, 7‐day, 30‐day	AUROC (NEWS):	7‐day mortality: NEWS – 6	NEWS score had good specificity and sensitivity for the prediction of 24‐h mortality
			24‐h mortality: 0.84 (95% CI, 0.82–0.86)		
			7‐day mortality: 0.81 (95% CI 0.80–0.82)		
			30‐day mortality: 0.76 (95% CI, 0.75–0.77)		
Ruan *et al*. (2016)	MEWS	90‐day mortality	AUROC (MEWS): 90‐day mortality: 0.883 (95% CI: not stated)	Mortality:	MEWS could be an effective tool to manage pre‐hospital emergency care
				Not reported	
				Critical illness:	
				MEWS – ≥4	
Saberian *et al*. (2022)	NEWS	In‐hospital mortality ICU admission	AUROC (NEWS):	Not reported	Difference between prehospital NEWS and ED ESI performance in predicting in‐hospital mortality and ICU admission was not significant.
			In‐hospital mortality: 0.82 (95% CI: 0.82–0.91)		
			ICU admission: 0.80 (95% CI: 0.71–0.89)		
Shaw *et al*. (2017)	NEWS	Discharged from ED, hospital ward admission, ICU admission, mortality (no timescale)	NEWS scores for patients who died in ED (7.20 ± 3.76; mean ± SD) or were admitted to ICU (7.46 ± 4.02) were higher than those admitted to a ward (3.13 ± 2.59) or discharged (1.72 ± 1.91).	Not reported	NEWS could successfully be utilised in the prehospital setting to predict those patients most likely to deteriorate.
Silcock *et al*. (2015)	NEWS	Mortality: 1‐day, 2‐day, 7‐day, 14‐day, 30‐day ICU admission within 48 h	AUROC (NEWS):	Recommendation of NEWS of 7	Elevated NEWS in prehospital pts is associated with a higher incidence of adverse outcomes
			1‐day mortality: 0.86 (95% CI: 0.69–1.0)		
			2‐day mortality: 0.87 (95% CI: 0.75–0.98)		
			7‐day mortality: 0.80 (95% CI: 0.70–0.89)		
			14‐day mortality: 0.79 (95% CI: 0.71–0.86)		
			30‐day mortality: 0.74 (95% CI: 0.66–0.82)		
			ICU admission: 0.77 (95% CI: 0.66–0.89)		

Mar‐Rodriguez *et al*. 2019a, 2019b, 2019c, 2020, 2021, 2023 – these studies had an overlapping period. The findings are not independent of each other, as the same patients are included across all/most studies.

† Adverse event: Defined as death, or requirement of immediate operative management, or admission to intensive care unit (ICU) or high dependency unit (HDU), or coronary care unit (CCU), or requirement of a medical team attendance, or transfer to a tertiary centre for definitive care.

‡ Clinical judgement: Pre‐alerting of hospitals by ambulance staff.

§ Time‐sensitive condition: The patient received a final diagnosis, and the condition required prompt prehospital management and limited waiting time at the hospital.

AUROC, area under receiver characteristic; CIP, critical illness prediction; DEPT, Danish emergency process triage; MEWS, Modified Early Warning Score; mNEWS, Modified National Early Warning Score; MREMS, Modified Rapid Emergency Medicine Score; NEWS, National Early Warning Score; NEWS2, National Early Warning Score 2; PI, Prehospital Index; TEWS, Triage Early Warning Score; ViEWS, Vitalpac Early Warning Score.

### Assessment of study quality and risk of bias

Study quality was assessed using a standardised critical appraisal instrument from the JBI Manual for Evidence Synthesis for Quasi‐experimental Studies.^19^ Included studies were assessed by two independent reviewers (DN and VT). Studies were graded as low risk for bias if they met all the criteria within the checklist, moderate risk of bias if they failed one of the criteria, and high risk for bias if they failed multiple criteria.

## Results

### Study selection

**Figure 1:**
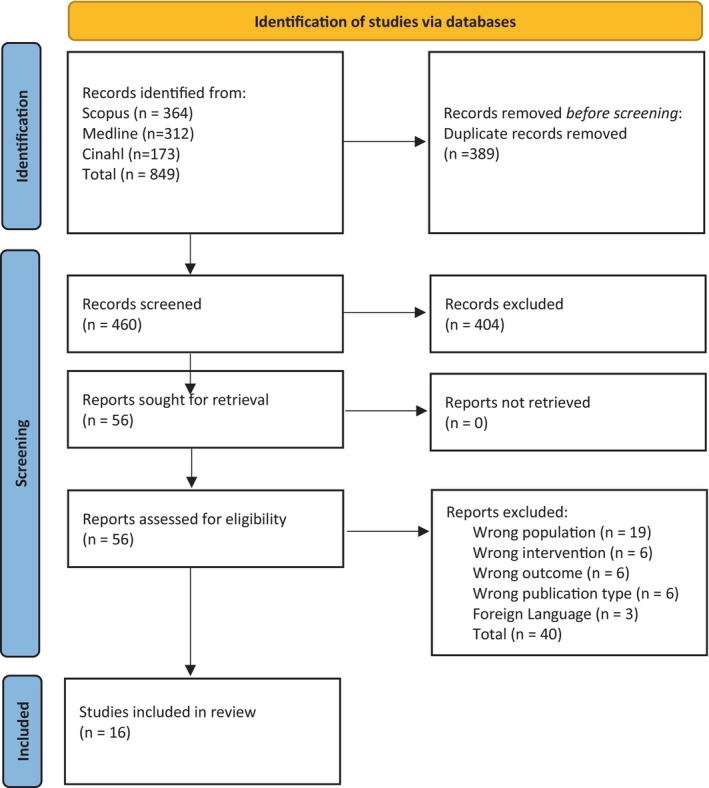
Preferred reporting items for systematic reviews and met‐analyses flow diagram.

Database searches identified a total of 849 citations. After the removal of duplicates, 460 remained. Based on a review of titles and abstracts, 56 papers were identified as potentially relevant. Of these, 16 relevant papers were then identified based on a review of full‐text articles (Fig. 1).

### Study characteristics

This systematic review identified 16 original research articles. An overview of the included studies is provided in Table 1. The research articles were published between 2012 and 2023, with six conducted in Spain,^1^, ^20^, ^21^, ^22^, ^23^, ^24^ three in the UK,^6^, ^25^, ^26^ two in Finland,^27^, ^28^ and one in Canada,^29^ China,^30^ Denmark,^31^ Iran^32^ and Sweden.^33^ The number of patients totalled 311 932, ranging from 287 to 121 837.

Nine studies were prospective observational studies,^1^, ^20^, ^21^, ^22^, ^23^, ^24^, ^30^, ^32^, ^33^ and seven were retrospective observational studies where EWS scores were calculated for the study on routinely collected data.^6^, ^25^, ^26^, ^27^, ^28^, ^29^, ^31^ One of these used a nested case–control design where EWS were only calculated for patients who had experienced an outcome of interest (ward, ICU admission, death in ED or discharge).^25^

Two studies only evaluated MEWS,^6^, ^30^ five studies only evaluated NEWS,^25^, ^26^, ^27^, ^28^, ^32^ and four studies only evaluated NEWS2.^1^, ^21^, ^22^, ^24^ Five studies compared a number of different EWS, which included MEWS, NEWS or NEWS2.^20^, ^23^, ^29^, ^31^, ^33^

The timing of the EWS calculation was not well reported. Nine studies specified that the first set of prehospital vital signs were used to calculate the EWS,^1^, ^6^, ^23^, ^24^, ^25^, ^27^, ^28^, ^29^, ^31^ three studies calculated EWS at different times in the prehospital journey,^24^, ^25^, ^31^ two studies indicated that the vital signs used were taken ‘on scene’ but did not specify if these were the first taken,^30^, ^33^ and five studies did not specify which prehospital vital signs were used^20^, ^21^, ^22^, ^26^, ^32^ (Table 1).

Various outcome measures were utilised to define severity: mortality (with timeframes ranging from 24 h to 90 days), ICU admission, critical illness, adverse events, and hospital admission. Although these outcome measures varied across studies, there was enough commonality to draw broad conclusions (Table 2).

### Risk of bias

This systematic review was conducted in accordance with the PRISMA guidelines. All included studies were assessed for methodological bias using the JBI critical appraisal checklist (Table 3).^19^ Five studies were considered to have a high risk of bias^25^, ^26^, ^29^, ^30^, ^32^ and 11 were considered to have a moderate risk of bias.^1^, ^6^, ^20^, ^21^, ^22^, ^23^, ^24^, ^27^, ^28^, ^31^, ^33^ No study had a low risk of bias. Although most studies considered confounding factors, only one study had a clear strategy to manage them.^6^

**TABLE 3:** JBI critical appraisal checklist for analytical cross‐sectional studies

Question	1	2	3	4	5	6	7	8	Risk of bias
Author									
Fullerton *et al*. (2012)	Yes	Yes	Yes	Yes	Yes	Yes	Unclear	Yes	Moderate
Hoikka *et al*. (2018)	Yes	Yes	Yes	Yes	Yes	No	Yes	Yes	Moderate
Lane *et al*. (2019)	Yes	Yes	Yes	Yes	Unclear	Unclear	Yes	Yes	High
Lindskou *et al*. (2023)	Yes	Yes	Yes	Yes	Yes	No	Yes	Yes	Moderate
Magnusson *et al*. (2020)	Yes	Yes	Yes	Yes	Yes	No	Yes	Yes	Moderate
Martin‐Rodriguez *et al*. (2019)	Yes	Yes	Yes	Yes	Yes	No	Yes	Yes	Moderate
Martin‐Rodriguez *et al*. (2019)	Yes	Yes	Yes	Yes	Yes	No	Yes	Yes	Moderate
Martin‐Rodriguez *et al*. (2019)	Yes	Yes	Yes	Yes	Yes	No	Yes	Yes	Moderate
Martin‐Rodriguez *et al*. (2020)	Yes	Yes	Yes	Yes	Yes	No	Yes	Yes	Moderate
Martin‐Rodriguez *et al*. (2021)	Yes	Yes	Yes	Yes	Yes	No	Yes	Yes	Moderate
Martin‐Rodriguez *et al*. (2023)	Yes	Yes	Yes	Yes	Yes	No	Yes	Yes	Moderate
Pirneskoski *et al*. (2019)	Yes	Yes	Yes	Yes	Yes	No	Yes	Yes	Moderate
Ruan *et al*. (2016)	Yes	No	Unclear	Unclear	No	No	Yes	Yes	High
Saberian *et al*. (2022)	Yes	Yes	Yes	Yes	No	No	Yes	Yes	High
Shaw *et al*. (2017)	Yes	Yes	Yes	Yes	No	No	Yes	Yes	High
Silcock *et al*. (2015)	Yes	Yes	Unclear	Yes	Yes	No	Yes	Yes	High

Questions:	Reviewer:
1. Were the criteria for inclusion in the sample clearly defined?	1. Dave Naylor
2. Were the study subjects and the setting described in detail?	2. Verity Todd
3. Was the exposure measured in a valid and reliable way	
4. Were objective, standard criteria used for measurement of the condition?	
5. Were confounding factors identified?	Rating System:
6. Were strategies to deal with confounding factors stated?	All “Yes” = Low risk of bias
7. Were the outcomes measured in a valid and reliable way?	A single “No” or “Unclear” = Moderate Risk of bias
8. Was appropriate statistical analysis used?	Greater than one “No′ or “Unclear” = High risk of bias

### Mortality

Mortality was reported in 15 of the 16 selected studies (Table 2). Timescales were measured from hospital admission and were reported in either ‘days’ or ‘hours’. It was unclear how a ‘day’ was defined. One study reported mortality but did not define a timescale.^25^ One study^6^ considered mortality as a contributing factor to a broader outcome of an ‘adverse event’.

Of the 15 studies that reported mortality, 13 used AUROC as a reporting statistic. The area under receiver operating characteristic curve (AUROC) provides a measure of the overall performance of a diagnostic test, in this case the diagnostic accuracy of EWS to predict mortality. For consistency of reporting, an AUROC of between 0.50 and 0.70 was considered to show a low diagnostic performance, an AUROC of between 0.70 and 0.80 a moderate performance, and an AUROC of between 0.80 and 0.90 a high diagnostic performance.^34^ The results ranged from 0.68 (95% CI: 0.64–0.73),^31^ suggesting low diagnostic performance, to 0.90 (CI 95%, 0.82–0.97),^20^ suggesting very good diagnostic performance. Overall, AUROC was generally higher for prediction of mortality in short timeframes (1 day, 24 h, 48 h), and lower for longer‐term mortality (30+ days; Table 2).

Hoikka *et al*.^27^ used positive likelihood ratios (PLR) and negative likelihood ratios (NLR) to show that a high‐risk NEWS score (≥9) was associated with a significantly higher probability of 1‐day mortality (PLR 17.36, NLR 0.21) and that a medium‐risk NEWS score was associated with a moderate increase in the probability of in‐hospital 1‐day mortality (PLR 4.58, NLR 0.14). Shaw *et al*.^25^ used ANOVA (analysis of variance) to show that an increasing NEWS score was associated with increasing severity of outcome (Table 2).

### ICU and hospitalisation

Four studies considered ICU admission as a discrete outcome,^24^, ^25^, ^26^, ^31^ and three of these used AUROC as a reporting statistic. The results ranged from 0.68 (95% CI: 0.67–0.69)^31^ to 0.76 (95% CI: 0.72–0.80),^24^ suggesting moderate diagnostic performance. One study^25^ used ANOVA to show that higher EWS was associated with ICU admission compared to admission to a hospital ward or hospital discharge. Only the study by Silcock *et al*.^26^ gave a time scale for ICU admission (48 h). Two studies^6^, ^33^ considered ICU admission as a contributing factor to a broader outcome: ‘adverse event’ and ‘time sensitive’ condition, respectively. It is therefore difficult to be clear about what contribution ICU admission made to these outcomes.

Only Shaw *et al*.^25^ considered hospital admission into a general ward as opposed to ICU admission, reporting that those patients who were admitted to hospital had a higher NEWS than those who were discharged (NEWS score of 2.6 *vs* 1.7).

### EWS *versus* current clinical practice

Only one study by Fullerton *et al*.^6^ looked at the relative performance of EWS compared with current clinical practice (non‐EWS). The primary outcome was an adverse event (death, immediate operative management, admission to ICU, requirement of emergency transfer team, or transfer to a tertiary centre for definitive care) within 24 h of hospital admission. The pre‐alerting of hospitals by ambulance staff was used as the indicator of critical illness recognition and, in the absence of any standardised protocol, relied on subjective criteria. The study had a relatively large sample size of 3504 patients and showed that implementing the MEWS increased sensitivity to an adverse event (death, immediate operative management, admission to ICU, requirement of emergency transfer team or transfer to a tertiary centre for definitive care) from 61.8% (95% CI, 51.0–72.8%) to 71.1% (95% CI, 61.1–81.6%), but decreased specificity from 94.1% (95% CI, 93.2–94.9%) to 76.2% (95% CI, 74.6–77.7%). An AUROC for MEWS of 0.80 (CI 95%, 0.74–0.86) demonstrated good diagnostic performance in predicting an adverse event.

### EWS timing

Three studies calculated EWS at different times in the prehospital patient journey. Shaw *et al*.^25^ calculated the EWS twice: on the first set of prehospital values and again on the last set of values before hospital admission, and found no significant difference between the initial and final NEWS calculation. Martín‐Rodríguez *et al*.^24^ calculated the EWS three times. The EWS based on the values taken on hospital arrival had significantly better performance for predicting 2‐day mortality (AUROC 0.94, 95% CI: 0.92–0.96) than both those based on the first set of prehospital values and those taken on evacuation from the scene (0.87, 95% CI: 0.83–0.91 and 0.90, 95% CI: 0.87–0.92, respectively). There was no significant difference between the performance for predicting mortality between the EWS based on the values taken on evacuation and on hospital arrival. Lindskou *et al*.^31^ also calculated the EWS three times, based on the first set of prehospital values, the last set of prehospital values, and the worst (highest scoring) set. The EWS based on the last set of prehospital values and the worst set of prehospital values had significantly better performance for predicting 1‐day mortality (AUROC 0.0.80, 95% CI: 0.79–0.81 and 0.76, 95% CI: 0.75–0.76, respectively) than those based on the first set of prehospital values (AUROC 0.72, 95% CI: 0.71–0.73). The same pattern was observed for 30‐day mortality and ICU admission.

### Comparative performance

Five studies compared the performance of different EWS systems.^20^, ^23^, ^29^, ^31^, ^33^ Although differences in design and settings make comparisons between these studies difficult, some broad themes can be identified. NEWS and NEWS2 had similar diagnostic performance for short‐term mortality and compared well against the other EWS systems included in these studies (Table 2). Two of these ‘comparative’ studies included the NEWS2 and MEWS,^20^, ^23^ with both showing that NEWS2 had a higher diagnostic ability for short‐term mortality than MEWS.

### Optimal cut‐off points for short‐term mortality

Seven studies considered what the optimal prehospital cut‐off point was for short‐term mortality (1 day, 24 h or 48 h).^1^, ^20^, ^21^, ^22^, ^23^, ^26^, ^28^ Two studies reported on MEWS, both giving an optimal cut‐off point of 5 (high‐risk, deteriorating patient)^20^, ^23^; four studies reported on NEWS, with optimal cut‐off points ranging from 7 to 12 (all high‐risk categories),^23^, ^26^, ^27^, ^28^ and four studies reported on NEWS2, with optimal cut‐off points ranging from 7 to 10 (all high‐risk categories).^1^, ^20^, ^21^, ^22^

## Discussion

This systematic review identified 16 studies that evaluated the use of prehospital EWS in predicting mortality, ICU admission and hospital admission. Different outcome measures were used across studies, and study designs and the cohort number varied; yet overall, current research tended to show that EWS had a potential application in the prehospital setting. All EWS reviewed generally had a good diagnostic performance in predicting short‐term mortality, with higher scores associated with increased mortality. This capacity decreased progressively in predicting mortality at 7 and 30 days. The EWS reviewed also had a moderate diagnostic performance in predicting ICU admission.

However, a previous review has suggested that prehospital EWS did not perform as well as EWS in the hospital setting for predicting mortality and proposed that this may reflect the higher acuity patient population found in hospital when compared with the prehospital patient population.^8^ In our systematic review, seven studies considered what the optimum prehospital MEWS, NEWS or NEWS2 cut‐off points were for predicting short‐term mortality.^1^, ^20^, ^21^, ^22^, ^23^, ^26^, ^27^ These optimal cut‐off points were all in the high‐risk categories (MEWS of 5, NEWS and NEWS2 ranging from 7 to 12), and are higher than those found to be optimal for in‐hospital EWS.^8^ As higher EWS scores target the more severely ill patients, these high cut‐off points suggest that prehospital EWS perform best in the prediction of clinical deterioration among the already critically ill.^31^ In the prehospital setting, these high EWS scores are reasonably uncommon with EWS scores generally being much lower than those calculated in hospitals.^8^ Therefore, the high cut‐off points used in the prehospital setting may be targeting a relatively small proportion of the general prehospital patient population. Lower cut‐off points often result in poor sensitivity and specificity in the prehospital setting.^31^

Six studies limited the responding crews to highly skilled advanced life support units.^1^, ^20^, ^21^, ^22^, ^23^, ^24^ These units generally respond to higher‐acuity patients, which may mean that these studies effectively selected a higher‐risk patient population. These studies found a significantly higher diagnostic performance for 48‐h/2‐day mortality (AUROC range 0.85–0.90) than a large study^31^ looking at the diagnostic performance for 1‐day mortality in an unselected population (AUROC 0.72, 95% CI: 0.71–0.73). This also suggests that the diagnostic ability of the EWS scores is outcome and patient population‐specific and may be limited in the prehospital setting.

It is important that EWS triage patients accurately. Several studies have suggested prehospital EWS tended to over‐triage patients.^21^, ^29^, ^31^ Although some degree of over‐triage may be necessary to limit potentially life‐threatening under‐triage, excessive over‐triage has wide‐ranging implications from resource inefficiencies to delayed care for critical patients.^35^ Insufficient sensitivity and specificity can also lead to under‐triage, with a low EWS score not necessarily meaning low risk.^1^, ^31^

The optimal timing of the EWS calculation in the prehospital setting remains unclear. In practical terms, if EWS can be incorporated into existing software systems, EWS could be calculated with every set of vital signs taken by the ambulance crew with very little increase in workload.^7^ This would also allow the responding crews to track EWS scores during transport.

### Strengths and limitations

This systematic review was conducted in accordance with the PRISMA guidelines.^15^ All included studies were assessed for methodological bias using the JBI critical appraisal checklist^19^ and included studies were assessed by two independent reviewers (DN and VT).

The studies in this review had significant heterogeneity in terms of populations, the timing of EWS, the definition of critical illness, ambulance dispatch, and prehospital treatment, which made comparisons difficult.

Three studies^20^, ^25^, ^36^ had very small sample sizes ranging from 189 to 349 patients, with the authors recognising the consequent poor utility of the statistical analysis and the need for a multicentre study with adequate power. Five studies appear to include the same participants within multiple studies.^1^, ^20^, ^21^, ^22^, ^23^, ^24^

For the retrospective studies, there was a high proportion of patients with incomplete datasets. This was managed inconsistently by either excluding these patients from the study, replacing the missing values with normal values, or using an imputation model. Many of the prospective studies did not have a clear approach to missing datasets with the assumption that this was not an issue due to the study design.

The timing of the EWS calculation was not recorded in five studies^20^, ^21^, ^22^, ^26^, ^32^ and recorded broadly as ‘on scene’ in two studies.^30^, ^33^ In all studies reviewed, it was unclear whether any treatment had occurred before the acquisition of vital sign measurements.

### Future research

All studies reviewed were considered to have a moderate or high risk of bias. The identification and management of confounding factors was a particular concern. An adequately powered prospective methodology investigating the ability of prehospital EWS to predict short‐term mortality may reduce the proportion of patients with incomplete datasets and increase the control of confounding factors such as patient populations, EWS timing, and treatment variations. It would also give the opportunity to investigate the effectiveness of EWS implementation (use of EWS tools and paramedic training packages) in prehospital practice.

There is currently very little research comparing the use of prehospital EWS to current practice. A retrospective cohort study investigating whether prehospital EWS has a higher diagnostic accuracy for predicting short‐term mortality compared with current practice (clinical judgement) would help establish whether prehospital EWS would improve patient outcomes.

## Conclusion

The literature indicated that, although EWS generally have good diagnostic accuracy in predicting short‐term mortality, the need to use relatively high EWS scores to identify at‐risk patients suggests that EWS may be less useful for unselected patient populations in the prehospital setting. The potential for under‐triage and over‐triage further limits EWS use. It also remains unclear whether the implementation of prehospital EWS would increase the identification of at‐risk patients and improve patient outcomes compared with clinical judgement. Therefore, there is currently insufficient evidence to recommend the use of prehospital EWS. EWS should not replace structured clinical evaluation and judgement but may be useful as a complementary and objective tool to aid the identification of high‐risk patients. Further research is necessary to determine if clinical judgement, when complemented by prehospital EWS, improves patient outcomes compared to approaches that do not utilise EWS.

## Data Availability

Data sharing not applicable to this article as no datasets were generated or analysed during the current study.

## References

[1] Martín‐Rodríguez F , López‐Izquierdo R , del Pozo Vegas C *et al*. Can the prehospital National Early Warning Score 2 identify patients at risk of in‐hospital early mortality? A prospective, multicenter cohort study. Heart Lung 2020; 49: 585–591. 10.1016/j.hrtlng.2020.02.047 32169257

[2] Kayambankadzanja RK , Schell CO , Wärnberg MG *et al*. Towards definitions of critical illness and critical care using concept analysis. BMJ Open 2022; 12: e060972. 10.1136/bmjopen-2022-060972 PMC9445819 36606666

[3] Patel R , Nugawela MD , Edwards HB *et al*. Can early warning scores identify deteriorating patients in pre‐hospital settings? A systematic review. Resuscitation 2018; 132: 101–111. 10.1016/j.resuscitation.2018.08.028 30171976

[4] Reay G , Rankin JA , Smith‐MacDonald L , Lazarenko GC . Creative adapting in a fluid environment: an explanatory model of paramedic decision making in the pre‐hospital setting. BMC Emerg. Med. 2018; 18: 1–11. 10.1186/s12873-018-0194-1 30442096 PMC6238402

[5] Bourke‐Matas E , Bosley E , Smith K , Meadley B , Bowles K‐A . Challenges to recognising patients at risk of out‐of‐hospital clinical deterioration. Australas. Emerg. Care 2023; 26: 24–29. 10.1016/j.auec.2022.07.003 35851506

[6] Fullerton JN , Price CL , Silvey NE , Brace SJ , Perkins GD . Is the Modified Early Warning Score (MEWS) superior to clinician judgement in detecting critical illness in the pre‐hospital environment? Resuscitation 2012; 83: 557–562. 10.1016/j.resuscitation.2012.01.004 22248688

[7] Williams TA , Tohira H , Finn J , Perkins GD , Ho KM . The ability of early warning scores (EWS) to detect critical illness in the prehospital setting: a systematic review. Resuscitation 2016; 102: 35–43. 10.1016/j.resuscitation.2016.02.011 26905389

[8] Guan G , Lee CMY , Begg S , Crombie A , Mnatzaganian G . The use of early warning system scores in prehospital and emergency department settings to predict clinical deterioration: a systematic review and meta‐analysis. PLoS One 2022; 17: e0265559. 10.1371/journal.pone.0265559 35298560 PMC8929648

[9] Nagarajah S , Krzyzanowska MK , Murphy T . Early warning scores and their application in the inpatient oncology settings. JCO Oncol. Pract. 2022; 18: 465–473. 10.1200/OP.21.00532 34995083

[10] Subbe CP , Kruger M , Rutherford P , Gemmel L . Validation of a Modified Early Warning Score in medical admissions. QJM 2001; 94: 521–526. 10.1093/qjmed/94.10.521 11588210

[11] Kyriacos U , Jelsma J , James M , Jordan S . Monitoring vital signs: development of a modified early warning scoring (MEWS) system for general wards in a developing country. PLoS One 2014; 9: e87073. 10.1371/journal.pone.0087073 24475226 PMC3901724

[12] Royal College of Physicians . National Early Warning Score (NEWS): Standardising the assessment of acute‐illness severity in the National Health Service. 2012.

[13] Royal College of Physicians . National early warning score (NEWS) 2: standardising the assessment of acute‐illness severity in the NHS. updated report of a working party. 2017.

[14] Fu L‐H , Schwartz J , Moy A *et al*. Development and validation of early warning score system: a systematic literature review. J. Biomed. Inform. 2020; 105: 103410. 10.1016/j.jbi.2020.103410 32278089 PMC7295317

[15] Page MJ , McKenzie JE , Bossuyt PM *et al*. The PRISMA 2020 statement: an updated guideline for reporting systematic reviews. Int. J. Surg. 2021; 88: 105906. 10.1136/bmj.n71 33789826

[16] Roland D , Powell C , Lloyd A *et al*. Paediatric early warning systems: not a simple answer to a complex question. Arch. Dis. Child. 2023; 108: 338–343. 10.1136/archdischild-2022-323951 35868852 PMC10176370

[17] Gerry S , Bedford J , Redfern OC *et al*. Development of a national maternity early warning score: centile based score development and Delphi informed escalation pathways. BMJ Med. 2024; 3: e000748. 10.1136/bmjmed-2023-000748 PMC11097818 38756669

[18] Ouzzani M , Hammady H , Fedorowicz Z , Elmagarmid A . Rayyan—a web and mobile app for systematic reviews. Syst. Rev. 2016; 5: 1–10. 10.1186/s13643-016-0384-4 27919275 PMC5139140

[19] Munn Z , Aromataris E , Tufanaru C *et al*. The development of software to support multiple systematic review types: the Joanna Briggs Institute System for the Unified Management, Assessment and Review of Information (JBI SUMARI). Int. J. Evid. Based Healthc. 2019; 17: 36–43. 10.1097/XEB.0000000000000152 30239357

[20] Martín‐ Rodríguez F , Castro‐ Villamor MÁ , del Pozo Vegas C *et al*. Analysis of the early warning score to detect critical or high‐risk patients in the prehospital setting. Intern. Emerg. Med. 2019; 14: 581–589. 10.1007/s11739-019-02026-2 30627928

[21] Martín‐Rodríguez F , López‐Izquierdo R , Del Pozo Vegas C *et al*. Accuracy of National Early Warning Score 2 (NEWS2) in prehospital triage on in‐hospital early mortality: a multi‐center observational prospective cohort study. Prehosp. Disaster Med. 2019; 34: 610–618. 10.1017/S1049023X19005041 31648657

[22] Martín‐Rodríguez F , López‐Izquierdo R , Del Pozo VC *et al*. A multicenter observational prospective cohort study of association of the prehospital national early warning score 2 and hospital triage with early mortality. Emerg. Med. Int. 2019; 2019: 5147808. 10.1155/2019/5147808 31355000 PMC6633971

[23] Martín‐Rodríguez F , Sanz‐García A , Medina‐Lozano E *et al*. The value of prehospital early warning scores to predict in‐hospital clinical deterioration: a multicenter, observational base‐ambulance study. Prehosp. Emerg. Care 2021; 25: 597–606. 10.1080/10903127.2020.1813224, 32820947 32820947

[24] Martín‐Rodríguez F , Sanz‐García A , Ortega GJ *et al*. Tracking the National Early Warning Score 2 from prehospital care to the emergency department: a prospective, ambulance‐based, observational study. Prehosp. Emerg. Care 2023; 27: 75–83. 10.1080/10903127.2021.2011995 34846982

[25] Shaw J , Fothergill RT , Clark S , Moore F . Can the prehospital National Early Warning Score identify patients most at risk from subsequent deterioration? Emerg. Med. J. 2017; 34: 533–537. 10.1136/emermed-2016-206115 28501815

[26] Silcock DJ , Corfield AR , Gowens PA , Rooney KD . Validation of the National Early Warning Score in the prehospital setting. Resuscitation 2015; 89: 31–35. 10.1016/j.resuscitation.2014.12.029 25583148

[27] Hoikka M , Silfvast T , Ala‐Kokko TI . Does the prehospital National Early Warning Score predict the short‐term mortality of unselected emergency patients? Scand. J. Trauma Resusc. Emerg. Med. 2018; 26: 48. 10.1186/s13049-018-0514-1 29880018 PMC5992854

[28] Pirneskoski J , Kuisma M , Olkkola KT , Nurmi J . Prehospital National Early Warning Score predicts early mortality. Acta Anaesthesiol. Scand. 2019; 63: 676–683. 10.1111/aas.13310 30623422

[29] Lane DJ , Wunsch H , Saskin R *et al*. Assessing severity of illness in patients transported to hospital by paramedics: external validation of 3 prognostic scores. Prehosp. Emerg. Care 2020; 24: 273–281. 10.1080/10903127.2019.1632998 31210571

[30] Ruan H , Zhu Y , Tang Z , Li B . Modified Early Warning Score in assessing disease conditions and prognosis of 10,517 pre‐hospital emergency cases. Int. J. Clin. Exp. Med. 2016; 9: 14554–14558.

[31] Lindskou TA , Ward LM , Søvsø MB , Mogensen ML , Christensen EF . Prehospital early warning scores to predict mortality in patients using ambulances. JAMA Netw. Open 2023; 6: e2328128. 10.1001/jamanetworkopen.2023.28128 37556138 PMC10413164

[32] Saberian P , Abdollahi A , Hasani‐Sharamin P , Modaber M , Karimialavijeh E . Comparing the prehospital NEWS with in‐hospital ESI in predicting 30‐day severe outcomes in emergency patients. BMC Emerg. Med. 2022; 22: 42. 10.1186/s12873-022-00598-5 35287593 PMC8922925

[33] Magnusson C , Herlitz J , Axelsson C . Pre‐hospital triage performance and emergency medical services nurse's field assessment in an unselected patient population attended to by the emergency medical services: a prospective observational study. Scand. J. Trauma Resusc. Emerg. Med. 2020; 28: 81. 10.1186/s13049-020-00766-1 32807224 PMC7430123

[34] Mandrekar JN . Receiver operating characteristic curve in diagnostic test assessment. J. Thorac. Oncol. 2010; 5: 1315–1316. 10.1097/JTO.0b013e3181ec173d 20736804

[35] Lokerman RD , Waalwijk JF , van der Sluijs R *et al*. Evaluating pre‐hospital triage and decision‐making in patients who died within 30 days post‐trauma: a multi‐site, multi‐center, cohort study. Injury 2022; 53: 1699–1706. 35317915 10.1016/j.injury.2022.02.047

[36] Semeraro F , Corona G , Scquizzato T *et al*. New early warning score: EMS off‐label use in out‐of‐hospital patients. J. Clin. Med. 2021; 10: 2617. 10.3390/jcm10122617 34198651 PMC8232239

